# The Dual Effects of Work Connectivity Behavior After-Hours on Employee Behaviors: Balancing Psychological Job Control and ICT Anxiety

**DOI:** 10.3390/bs15060796

**Published:** 2025-06-10

**Authors:** Lijun Chen, Shimin Zhang

**Affiliations:** School of Public Affairs, Zhejiang University, Hangzhou 310058, China; lijunchen@zju.edu.cn

**Keywords:** work connectivity behavior after-hours, in-role behaviors, extra-role behaviors, psychological job control, ICT anxiety

## Abstract

The dual effects of work connectivity behavior after-hours (WCBA) on employees’ in-role and extra-role behaviors were investigated using the framework of the Job Demands–Resources (JD-R) model. A two-wave cross-sectional design with a one-week interval was employed, and data were acquired from a survey of 402 Chinese employees. The results showed that WCBA positively influenced in-role and extra-role behaviors through enhanced psychological job control (β = 0.1908 and β = 0.1356, respectively), while also exerting negative effects via increased ICT anxiety (β = −0.0190 and β = −0.0434, respectively). The findings indicate that although WCBA can foster work outcomes through increased job control, it also carries the risk of undermining these benefits due to the psychological strain from ICT-related stress. Therefore, organizations should support employees in managing WCBA effectively by balancing potential productivity gains with an awareness of its psychological costs. This research uniquely provides a simultaneous investigation of both behavior types within a unified dual-pathway framework based on the Job Demands–Resources (JD-R) model. This research also extends our knowledge of the nuanced influence of after-hours connectivity and has potential application in the optimization of the performance and welfare of employees in digitally connected work environments.

## 1. Introduction

With the rise of the digital economy, organizations are extensively utilizing emerging information technologies, leading to profound changes in employees’ work patterns. Work is no longer restricted to specific locations. Employees now have the flexibility to utilize smart devices and digital software to work and communicate outside of office hours. Work gradually encroaches into employees’ non-work domains, leading to work connectivity behavior after-hours (WCBA). WCBA, as a representative new form of work, has significantly impacted employee behaviors (Chu et al., 2024). Although WCBA can enhance the proactive actions of employees by increasing their sense of control, flexibility, and autonomy at work (Haynes, 2008), the widespread application of information and communication technology creates an ever-connected environment, requiring employees to respond and use electronic devices for work during their personal time. In the Chinese context, state-owned enterprises typically consider information and communication technology as a core enabler of digital transformation, primarily applied to process automation across document workflows, financial sharing systems, and asset management. It is a common practice for employees to conduct work-related interactions beyond official working hours using organization platforms or widely used apps such as WeChat and DingTalk.

This phenomenon may cause pressure on employees, leading to ICT anxiety, emotional fatigue, and burnout, and influencing in-role and extra-role behaviors (J. Li et al., 2024; Yang et al., 2022). The former is related to job responsibilities, including completing work tasks and complying with rules and regulations, and directly affects organizational performance (Kim & Mauborgne, 1996). Although extra-role behaviors are not linked directly to job responsibilities, they can indirectly impact organizational performance and atmosphere (Tastan & Davoudi, 2015; Hsu et al., 2017). Extra-role behaviors often manifest through informal after-hours efforts enabled by WCBA, such as proactive collaboration or team support through the use of digital platforms. These discretionary actions, although not formally required, are often culturally expected and reinforced by organizational norms in China (Lin et al., 2020). Both types of behavior are critical in assessing employee performance and enhancing organizational effectiveness (Kim & Mauborgne, 1996). The present environment offers a unique opportunity to evaluate the effects of WCBA on these behaviors, as well as to improve and enhance its advantages within organizations in the future.

Especially during the COVID-19 pandemic, as employees began working from home, WCBA has gradually attracted scholars’ attention (Isabell et al., 2023; Vaiman et al., 2021). Nevertheless, the effect of WCBA on in-role and extra-role behaviors remains unclear (Palumbo et al., 2023). There is a lack of consensus in study findings, with some studies reporting that it positively impacts employees’ engagement with work, thriving, and innovative behaviors (Carmeli & Spreitzer, 2009; Yang et al., 2023), whereas others have concluded that the impact is negative (Luna-Arocas et al., 2020; Rennecker & Godwin, 2005; Yao et al., 2024). These discrepancies in the impact of both WCBA and remote working, in general, have fueled the controversy surrounding the “bright” and “dark” aspects of this new type of work arrangement (Kraus et al., 2023). It is, thus, necessary to evaluate “how” the positive and negative effects of WCBA are generated and how they influence in-role and extra-role behaviors.

To date, however, most investigations of WCBA have focused on its impact on conflict between work and family (X.-C. Yu et al., 2019; Dong et al., 2022), as well as its separate effects on in-role and extra-role behaviors (Nurmi & Hinds, 2020; Yang et al., 2023). Prior studies have overlooked significant issues, such as the changes WCBA introduces to work and the surrounding environment, which can both influence behaviors. Despite widespread observations of these changes, they have been largely neglected in the literature (Guest, 2017). The impact of WCBA on these behaviors should be considered simultaneously. Encouraging and supporting employees to show positivity in these behaviors can favorably influence organizational performance (Tremblay et al., 2010). On the one hand, understanding whether employees demonstrate additional effort and contributions while completing core job tasks is essential for a comprehensive performance evaluation (Becker & Kernan, 2003).

On the other hand, a simultaneous focus on both behavior types would improve organizations’ understanding of the overall contributions of the employees (Hui et al., 1999). Therefore, this study first evaluated the influence of WCBA on in-role and extra-role behaviors, followed by an examination of the associated mechanisms.

These issues were addressed using the Job Demand–Resource (JD-R) framework to evaluate the reactions of individuals to WCBA and their behavioral consequences. The JD-R model suggests that job-related factors can be classified into two categories based on their impact on individual resources: job resources and job demands (Demerouti et al., 2001). Adequate resources form the foundation for individual work engagement and proactive behavior, effectively stimulating employees’ intrinsic motivation. When job demands exceed an individual’s available resources, it can lead to negative perceptions and emotional distress among employees (Zhu et al., 2024).

WCBA is considered a job resource that enhances employees’ perceptions of control over work time and location by granting them flexibility and autonomy (Van Zoonen et al., 2023), influencing their specific behaviors (Parker & Collins, 2010; Ten Brummelhuis et al., 2021). Conversely, a perception of WCBA as an additional demand of the job can have negative consequences if employees feel overwhelmed (Demerouti et al., 2001). WCBA is linked to the need to utilize information and communication technology (ICT) for processing work-related information, which can further lead to emotional demands and negatively influence behavior (H. A. Richardson et al., 2021). Thus, WCBA may represent a double-edged sword. Therefore, this study sought to address the following research question: How does work connectivity behavior after-hours (WCBA) influence employees’ in-role and extra-role behaviors through the dual mechanisms of psychological job control and ICT anxiety, as conceptualized based on the JD-R model?

This investigation aimed to add to the current knowledge of WCBA. Unlike previous investigations that have addressed in-role and extra-role behaviors separately, this research provides a simultaneous assessment of the effects of WCBA on both behavior types. Second, the theoretical base of the JD-R model was utilized to assess the mediating effects of psychological job control and ICT anxiety. Third, the research findings are anticipated to provide organizations with valuable insights into understanding employee behaviors, offering timely support and assistance, and building harmonious labor relations.

## 2. Theoretical Context and Hypothesis Development

### 2.1. In-Role and Extra-Role Behaviors and WCBA

Workplace behavior is typically divided into in-role and extra-role behaviors (Rotundo & Sackett, 2002). The former category includes behaviors necessary to accomplish job responsibilities, including those that are required, assessed, and rewarded by the organization and are subordinate to employee work (Williams & Anderson, 1991), which can effectively influence an organization’s performance. This study considers in-role behaviors to be those that are formal and explicitly stated in the job description. Previous research has studied the antecedent influences on in-role behaviors, such as psychological contract fulfillment (Turnley et al., 2003), ethical work climate (Leung, 2008), organizational commitment (C. C. Huang & You, 2011), leadership (J. T. Huang & Hsieh, 2015), and others (Taamneh et al., 2024; Nwanzu & Babalola, 2024). With the continuous development of emerging information technologies, factors such as WCBA related to technology (Cheng et al., 2022) may potentially impact in-role behaviors. The emergence of WCBA demonstrates the gradual tendency to blur the boundaries between work and non-work, with work-related issues being carried over into non-working hours. This phenomenon potentially influences their in-role behaviors. Yang et al. (2023) indicated that WCBA has a positive influence on engagement with work by enhancing autonomy, thereby increasing the positive impact on in-role behaviors. Fonner and Roloff (2010) highlighted the reduction in work–family conflict and emotional fatigue during remote working, while Fujimoto et al. (2016) demonstrated that WCBA is associated with autonomy and work continuity. However, J. Li et al. (2024), using the Conservation of Resources theory, found that WCBA leads to negative effects on in-role behaviors by increasing employees’ feelings of alienation and distress, which subsequently cause them to engage in non-work-related activities during working hours. Therefore, it is important for organizations to appreciate the potential effect of WCBA on in-role behaviors and take appropriate measures to promote employees’ work–life balance, thereby ensuring optimal working performance.

Extra-role behaviors refer to conscientious and voluntary behaviors that are beneficial to or aimed at benefiting the organization but go beyond the organization’s expectations for existing roles (Van Dyne et al., 1995). In the context of widespread WCBA, such extra-role behaviors often occur outside of official working hours. For example, employees may voluntarily help colleagues resolve urgent issues, engage in after-hours communication to support team tasks or share work-related knowledge via digital platforms such as WeCom (enterprise WeChat) or DingTalk. Previous research focuses more on extra-role behaviors in several main areas, including the dimensions and measurement of various types of behaviors within the category, the impact of these behaviors or specific behaviors on organizational performance, and the factors and mechanisms influencing employees’ extra-role behaviors. For example, some studies found that a perception of learning environments encourages individuals to be proactive, creative, and willing to share knowledge (Islam & Tariq, 2018), concluding that resource-seeking influences extra-role behaviors (Demerouti et al., 2015). WCBA, as a specific work arrangement associated with advances in communication technology, represents a product of a unique new working situation. Liu et al. (2023) found that WCBA not only increased the unethical pro-family activities of employees (such as misusing organizational resources for personal family matters) but could also promote such activities indirectly via work–family conflict, thereby increasing the negative impact on extra-role behaviors. H. He et al. (2023) reported similar findings, demonstrating that both proactive and passive WCBA can negatively affect family harmony. In contrast, researchers who have observed a positive relationship argue that it can be explained by the advantages of digital connectivity after hours, mainly enhanced performance resulting from reduced emotional exhaustion (Ren et al., 2023). By investigating how WCBA influences employees’ extra-role behaviors, organizations can gain insights into employees’ psychological states and emotional changes. It is thus important to determine the mechanism by which WCBA influences employees’ extra-role behaviors.

Although previous research has focused exclusively on whether WCBA positively or negatively impacts job performance (C. He & Sun, 2023; Cheng et al., 2022; Ten Brummelhuis et al., 2021), our understanding of how WCBA simultaneously influences both in-role and extra-role behaviors and through what mechanisms remains limited. This study addresses the gap in research by applying the JD-R framework to explore two parallel mechanisms. In the JD-R model, job features can be classified as demands and resources (Demerouti et al., 2001). Here, the former represents “negative factors” that consume energy, while the latter refers to “positive factors” that promote the realization of goals, learning, growth, and development in a job. Accordingly, we examined the underlying mechanisms through which WCBA influences employee behavior, utilizing the JD-R framework in terms of both positive (resource enhancement via psychological job control) and negative (resource depletion via ICT anxiety) pathways.

### 2.2. The Mediating Effect of Psychological Job Control

Psychological job control is a psychological construct that reflects individuals’ perceived control over the timing and location of task execution in the workplace (Kossek et al., 2006). This has been shown to be positively influenced by WCBA (K. Richardson & Thompson, 2012). Abdeen and Khalil (2023) further demonstrated that digital connectivity contributes positively to employee outcomes, suggesting that connectivity may function as a job resource rather than a strain. WCBA enables employees to respond to and manage work tasks at flexible times and locations, helping them to achieve psychological job control over their work progress (Mazmanian et al., 2013) and improve the control employees have over their environment (Haynes, 2008). WCBA also satisfies employees’ psychological need for autonomy and serves as a source of psychological job resources (Xanthopoulou et al., 2007).

Recent studies have also shown that WCBA can serve as a valuable job resource by enhancing employees’ autonomy and sense of control over their work tasks. Y. Li et al. (2025) proposed an updated JD-R 3.0 framework wherein after-hours connectivity facilitates psychological job control by allowing employees to choose when and how to complete their responsibilities. Similarly, Chu et al. (2024) emphasized that work connectivity in digital contexts enables proactive behaviors by giving employees increased flexibility and responsiveness in managing their performance. Therefore, WCBA can empower employees to take ownership of their time and workflow, reinforcing the motivational benefits of psychological job control. The perception of WCBA as an important resource instead of an added burden enhances communication and permeability between various work-associated areas, allowing for more frequent resource flow between fields (Duxbury et al., 2014).

The JD-R model (Kwon & Kim, 2020) helps to elucidate the effect of WCBA on in-role behaviors. Control is one of the four experiences promoting recovery (Sonnentag & Fritz, 2007). As a new form of job control, psychological job control can actively facilitate employees’ recovery processes (Steed et al., 2021), thereby better replenishing the resources consumed during work. Consistent with this model, Bakker and Demerouti (2017) noted that psychological job control is considered an energy resource that is valuable for assisting employees in further acquiring other necessary resources. Obtaining the necessary resources increases the likelihood of employees investing their energy and time in responsibilities and duties that require significant resource consumption, thus allowing them to fulfill their in-role behaviors more efficiently. Moreover, when employees perceive their ability to control work time and location, they become more motivated and more effective in completing their assigned tasks (Daniels & Guppy, 1994). Therefore, we hypothesize the following:

**Hypothesis** **1.** *Psychological job control mediates the association between WCBA and in-role behaviors.*

Organizations frequently want their employees to extend performance beyond formally required activities (Griffin et al., 2007; Welbourne & Paterson, 2017). Psychological job control encourages employees to shift their focus from formal work tasks to organizationally valuable extra-role behaviors (Bond & Flaxman, 2006). WCBA has created a new work pattern, enhancing individuals’ sense of autonomy in their work. Individuals can choose to complete tasks at any time and from any place after working hours, reducing excessive supervision from managers and unnecessary interruptions from colleagues. This flexibility enables employees to perform actions that enhance organizational development beyond their assigned duties (Sulea et al., 2012). In the JD-R model, WCBA helps employees break free from temporal and spatial constraints, enhancing their perception of autonomy and discretion in work decisions, which can be considered an increase in resources (Fujimoto et al., 2016). Increased availability of resources can stimulate employees’ intrinsic motivation, enabling them to maintain work vitality while surpassing organizational expectations. Furthermore, employees with ample resources are better equipped to acquire resources and initiate a positive gain spiral (Hobfoll, 2001), which encourages further engagement in extra-role behaviors that generate added value to the group or organization. Accordingly, we hypothesize the following:

**Hypothesis** **2.** *Psychological job control mediates the association between WCBA and extra-role behaviors.*

### 2.3. The Mediating Influence of ICT Anxiety

ICT is an electronic device or technology capable of collecting, storing, or transmitting information (Day et al., 2012). Based on the perspectives of Hsieh et al. (2020), ICT anxiety is described here as feelings of discomfort when utilizing technology and hesitancy in adopting new technology. Advances in ICT have revolutionized work, enabling connections between employees and the office and continuous communications with no restrictions on space and time (Piszczek, 2017). WCBA results in the utilization of ICT for work outside working hours, invading employees’ personal time and life domains with work-related demands (Park et al., 2011). Therefore, personal time and other resources are compromised, leading to delayed recovery experiences and increased susceptibility to emotions such as ICT anxiety (Beaudry & Pinsonneault, 2010). Employees proactively and frequently check their mobile communication devices to avoid missing information (Przybylski et al., 2013) and always stay on high alert for information, which further contributes to ICT anxiety (C. Wang et al., 2023).

Czaja et al. (2006) explained that individuals who experience anxiety about using technology are less likely to use it, indicating ICT anxiety can negatively affect technology acceptance and use. Ellis and Allaire (1999) also argued that ICT anxiety leads individuals to be unwilling to utilize technology. Both perspectives are related, as ICT anxiety can influence an individual’s choice to utilize technology (Meuter et al., 2003), largely affecting their attitude (Celik & Yesilyurt, 2013). Given that WCBA requires the use of ICT, ICT anxiety can reduce an individual’s work efficiency and negatively impact in-role behaviors. Saadé and Kira (2009) confirmed this view, and they also suggested that ICT anxiety affects welfare and social relationships. Moreover, the ICT anxiety generated by using ICT to handle and receive work-related material outside of working hours can induce a sense of interruption, potentially diverting attention from the task (Derks et al., 2021) and negatively impacting in-role behaviors. Thus, the following is proposed:

**Hypothesis** **3.** *ICT anxiety mediates the association between WCBA and in-role behaviors.*

As discussed by Prodanova and Kocarev (2021), the utilization of technology is also associated with job demands and resources. Anxiety related to technology use (a job demand) can affect employees’ job performance (Suryanto et al., 2022). In the JD-R model, individuals consistently endeavor to amass and protect resources, and those without resources are more vulnerable to emotional stress caused by resource loss, accelerating their descent into a spiral of resource depletion (Bakker et al., 2014). The ICT anxiety caused by WCBA leads to self-depletion among employees. When individuals perceive work-related threats and feel unable to deal with them, they reduce their resource investment in extra-role behaviors and increase their resource investment in self-protection as a defense and counteractive mechanism (Jesus et al., 2019). S. Yu et al. (2021) found that anxiety can reduce the extra-role behaviors of employees. Hypothesis 4 is thus proposed as follows:

**Hypothesis** **4.** *ICT anxiety mediates the association between WCBA and extra-role behaviors.*

**Figure 1:**
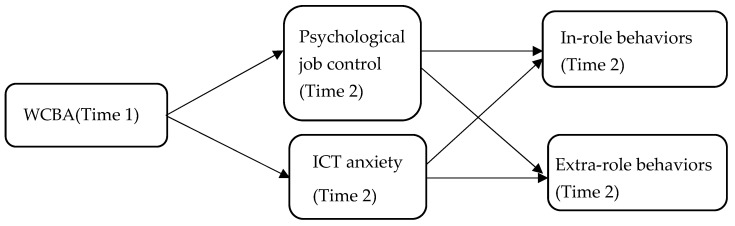
Research model. Note(s): WCBA = Work connectivity behavior after-hours, ICT anxiety = Information and communication technology anxiety. Source(s): Authors’ creation/work.

The model of the study is illustrated in Figure 1.

## 3. Methodology

### 3.1. Participants and Data Collection

This study focused on employees working in Chinese state-owned enterprises (SOEs) who typically work on-site. However, due to the administratively driven nature of SOE operations, a culture of overtime persists, especially during periods involving government-mandated projects or critical performance evaluation cycles. With the growing adoption of information and communication technologies (ICTs), most SOEs have implemented enterprise-level digital collaboration platforms. These systems enable employees to access work-related content through mobile devices (e.g., smartphones, tablets, laptops) and engage in work tasks beyond regular working hours via organization platforms.

A questionnaire was constructed in accordance with previous studies. The questionnaire was first written in English, followed by a Chinese translation using a back-translation method to maintain the equivalence of the concepts (Brislin, 1970). The questionnaire was re-tested on 50 individuals for verification and refinement.

Completion of the questionnaire was performed online and was both voluntary and anonymous (F. Wang et al., 2023). The Credamo platform (https://www.credamo.com, accessed on 11 September 2023) was utilized for the distribution of questionnaires; this platform resembles Amazon Mechanical Turk and is used in China for data collection. It is recognized by numerous international social science and public administration journals (Fu et al., 2020).

This research employed a two-wave cross-sectional design with a one-week interval. First, the WCBA and sociodemographic information scales were sent to the employees working in the state-owned enterprises. One week later (timepoint 2), these employees completed the sections on psychological job control, ICT anxiety, and in-role and extra-role behaviors. The first phase collected 600 questionnaires, while the second collected 472 questionnaires. To ensure data quality, two quality control procedures were used to identify and remove invalid questionnaires. First, we screened for patterned responses, including instances where respondents selected answers in highly regular sequences (e.g., repeatedly selecting the same option or rotating through 1–2–3). Second, we included an attention-check item (“Please select number 1 for this item”) to detect inattentive responses. Questionnaires failing this item or showing highly patterned responses were excluded from the final dataset. Following the removal of blank or invalid questionnaires, 402 surveys were included in the analysis, representing an 85.17% effective response rate. Table 1 provides the sociodemographic details of the participants.

### 3.2. Measures

WCBA: This scale comprised 7 items, which were adapted from K. Richardson and Benbunan-Fich (2011). Two aspects were assessed, namely, connectivity duration (time spent using mobile phones, tablets, and computers) and frequency (how often mobile phones, tablets, and computers were used after working hours). As described by Dong et al. (2022), responses were collected for 4 time periods, namely, before and after working hours, during off-days, and over the weekend or vacation. Responses were given as minutes, such as 1–15 min and 16–30 min. The Cronbach’s alpha of the questionnaire was 0.713. WCBA frequency was assessed according to Boswell and Olson-Buchanan (2007). Furthermore, as noted by Dong et al. (2022), participants described the frequency of using mobile phones, tablets, and computers in specific non-work activities, such as during shopping, meals, or traveling. Responses were averaged to construct the overall WCBA frequency index. The Cronbach’s alpha values were 0.779 and 0.782 for the overall scale.

The scales used for the following variables—psychological job control, ICT anxiety, in-role behaviors, and extra-role behaviors—were adapted from earlier studies. A 5-point Likert scale was used, ranging from 1 (strongly disagree) to 5 (strongly agree).

Psychological job control: A 4-item scale was utilized, as described by Kossek et al. (2006). Sample questions included, “To what extent does your job permit you to decide on the location of your work?” and” How much autonomy is on your job?”. The Cronbach’s alpha was 0.813.

ICT anxiety: This scale comprised 4 items adapted from Van Raaij and Schepers (2008). Examples include, “I am hesitant to use a computer as I am afraid of making mistakes I am unable to correct,” and “Computers make me feel uneasy”. The Cronbach’s alpha was 0.832.

In-role behaviors: These were assessed with 7 items, as described by Williams and Anderson (1991). Examples include, “I complete assigned tasks adequately”, ”I meet formal performance requirements of the job”, and ”I fulfill my basic duties”. The Cronbach’s alpha was 0.844.

Extra-role behaviors: Following Williams and Anderson (1991), 14 items were assessed. Examples include, “I help others who are experiencing heavy workloads”, “I take time to listen to co-workers’ problems and worries”, and “I help others who have been absent”. The Cronbach’s alpha was 0.911.

Control variables: These possible confounders included gender, age, education, position, and tenure based on the previous literature. Sociodemographic variables such as gender, age, and educational level are typically utilized (Liu et al., 2023). Vadera et al. (2013) reported a marked association between educational level and extra-role behaviors. Position and tenure were also utilized as control variables (Dong et al., 2022).

### 3.3. Reliability and Validity

The discriminant validity of the model was assessed using confirmatory factor analysis, indicating a good fit between the measurement model and the data (χ^2^/df = 1.739, RMSEA = 0.043, CFI = 0.946, TLI = 0.936, SRMR = 0.0589). Thus, the model demonstrated good discriminant validity (see Table 2).

### 3.4. Common Method Bias

Although the data in this study were collected at two different time points, the use of self-report measures may still raise concerns about common method bias. Therefore, both procedural and statistical remedies were applied to mitigate its potential impact. Procedurally, we adopted several techniques, including time-lagged data collection, anonymous responses, and the inclusion of attention-check items. Statistically, this was assessed with Harman’s one-factor test (Podsakoff & Organ, 1986). The first factor explained 29.28% of the variance (<40%), indicative of no marked CMB. The goodness-of-fit indices for the one-factor model (Table 2) were χ^2^ = 4222.994, df = 598, IFI = 0.501, CFI = 0.499, and RMSEA = 0.123, whereas those of the five-factor model were χ^2^ = 921.739, df = 530, IFI = 0.947, CFI = 0.946, and RMSEA = 0.043. The results indicate that the five-factor model is superior to the one-factor model, indicating a low CMB.

## 4. Results

Data on the variables and correlations are provided in Table 3. The variance inflation factor (VIF) was assessed in advance, indicating that all values for the explanatory variables were <3. The indicators of the control variables ranged between 1.042 and 2.611, all below the threshold of <3 (Black et al., 2010), demonstrating minimal multicollinearity.

**Figure 2:**
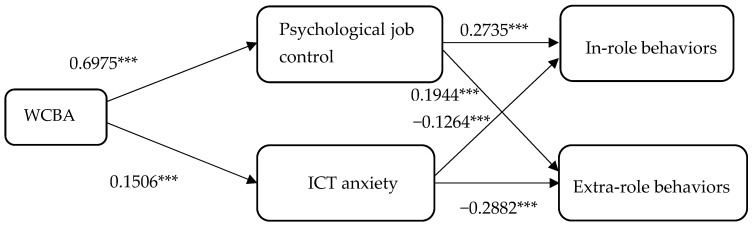
Model path coefficient. Note(s): *** *p* < 0.01. Source(s): Authors’ creation/work.

Following the method proposed by Preacher and Hayes (2008) for testing mediation effects, we used the PROCESS macro along with the bootstrap method to test the mediating effect of psychological job control on ICT anxiety. Following Hayes (2012), we utilized Model 4, representing a simple mediation model. Bootstrap resampling was conducted 5000 times, controlling for gender, age, education, position, and tenure. The results of the path analysis are presented in Table 4 and Figure 2. After controlling for gender, age, education, position, and tenure, the explained variance (R^2^) of the predictive models for the outcome variables is reported in Table 5, and the indirect effects are presented in Table 6.

Based on the results, WCBA is significantly and positively associated with psychological job control (β = 0.6975, *p* < 0.01), and psychological job control positively predicts in-role behaviors (β = 0.2735, *p* < 0.01). The indirect effect of WCBA on in-role behaviors through psychological job control is 0.1908, with a 95% confidence interval of [0.1274, 0.2724]; this interval does not include 0, indicating that psychological job control serves as a mediator in this relationship. Therefore, Hypothesis H1 is supported. In addition, psychological job control positively predicts extra-role behaviors (β = 0.1944, *p* < 0.01). The indirect effect of WCBA on extra-role behaviors through psychological job control is 0.1356, with a 95% confidence interval of [0.2329, 0.591], which also excludes 0. This suggests that psychological job control mediates this relationship as well, supporting Hypothesis H2.

WCBA is also significantly and positively associated with ICT anxiety (β = 0.1506, *p* < 0.01), whereas ICT anxiety negatively predicts in-role behaviors (β = −0.1264, *p* < 0.01). The indirect effect of WCBA on in-role behaviors via ICT anxiety is −0.019, with a 95% confidence interval of [−0.0407, −0.0045], which excludes 0, indicating that ICT anxiety functions as a mediator in this relationship. Thus, Hypothesis H3 is supported. Furthermore, ICT anxiety negatively predicts extra-role behaviors (β = −0.2882, *p* < 0.01). The indirect effect of WCBA on extra-role behaviors via ICT anxiety is −0.0434, with a 95% confidence interval of [−0.0731, −0.0174], which also excludes 0. This confirms the mediating role of ICT anxiety, supporting Hypothesis H4.

To clarify the nature of the mediation effects, we examined the total, direct, and indirect effects of WCBA on in-role and extra-role behaviors. The total effect of WCBA on in-role behaviors was β = 0.5118, and that on extra-role behaviors was β = 0.3669. After separately controlling for psychological job control and ICT anxiety, the direct effect of WCBA on in-role behaviors (β = 0.3211 and β = 0.5309, respectively) as well as that on extra-role behaviors (β = 0.2313 and β = 0.4103, respectively) remained significant, indicating partial mediation in both models. The indirect effects of psychological job control were β = 0.1908 (in-role) and β = 0.1356 (extra-role), while those of ICT anxiety were β = −0.0190 and β = −0.0434, respectively. Bootstrap analysis revealed that the indirect effects of WCBA on in-role and extra-role behaviors through enhanced psychological job control (β = 0.1908 and β = 0.1356, respectively) were significantly stronger than the negative indirect effects through ICT anxiety (β = −0.0190 and β = −0.0434, respectively). These results suggest that, in practice, the positive impact of enhanced psychological control may partially offset the negative impact of ICT anxiety. This conclusion aligns with the theoretical framework of the JD-R model, which posits that the motivational benefits of job resources may outweigh the detrimental effects of job demands (Bakker & Demerouti, 2017; Demerouti et al., 2001; Hobfoll, 2001).

These findings support the dual-path theoretical model, demonstrating that WCBA functions simultaneously as a job resource—by enhancing psychological control, which promotes both in-role and extra-role behaviors—and as a job demand—by increasing ICT-related strain, which impairs these behaviors.

## 5. Discussion

Our findings indicate a positive association between WCBA and psychological job control, promoting both in-role and extra-role behaviors. This finding agrees with earlier research that WCBA offers greater job resources to employees, such as flexibility (Diaz et al., 2012) and work engagement (Yang et al., 2023), to accomplish in-role behaviors. Additionally, WCBA can enhance employees’ control over work progress (Haynes, 2008), thereby improving efficiency and facilitating employees to take part in extra-role behaviors.

Second, this study not only supports the positive view but also rationalizes the negative effects of WCBA, wherein WCBA increases employees’ ICT anxiety, consequently diminishing their in-role and extra-role behaviors. In fact, prior research has also hinted at the adverse effects of WCBA. For instance, Chen and Casterella (2018) found that WCBA can lead to emotional exhaustion among employees, resulting in adverse effects. Additionally, WCBA can decrease employees’ work engagement (Barley et al., 2011; Igbaria & Guimaraes, 1999), thus negatively affecting their behaviors. Moreover, Wei (2024) found that WCBA can increase employees’ job stress, thereby triggering their withdrawal behaviors at work. Our results reveal the cost of WCBA, consistent with findings on digital fatigue and ICT strain (Derks et al., 2021). This is different from previous studies that exclusively considered general anxiety. This research focuses on the mediating variable of ICT anxiety in the negative pathway, which is closely associated with the context of WCBA.

Utilizing the JD-R model, this research incorporates both the positive effect (enhancing psychological job control) and the negative effect (increasing ICT anxiety) of WCBA into the theoretical model. This study contributes to the literature by applying the JD-R model to uncover the dual mechanisms through which WCBA affects employee behaviors. Specifically, it demonstrates that WCBA has both resource-enhancing effects—by improving psychological job control—and resource-depleting effects—by increasing ICT anxiety. These two mediators operate simultaneously, reflecting the dual-pathway framework of the JD-R model. By showing that WCBA can foster in-role and extra-role behaviors through increased job control while simultaneously diminishing them through ICT-induced stress, this research offers a more nuanced theoretical understanding of how work connectivity after-hours influences workplace outcomes. This dual mediation perspective provides deeper insight into the conflicting outcomes associated with digital work environments, moving beyond simplistic positive or negative interpretations of WCBA. Moreover, by linking WCBA with both in-role and extra-role behaviors in a unified framework, this study extends prior JD-R applications that typically examine work outcomes in isolation. The results of this study align with the JD-R framework’s assertion that workplace characteristics can operate through both motivational and strain pathways. From an integrative perspective, we propose that WCBA represents a “double-edged sword”.

### 5.1. Theoretical Contributions

Our first contribution to current knowledge on in-role and extra-role behaviors is from the perspective of job resources and demands to explore their antecedents. Previous research has primarily focused on the effects of role conflict, role ambiguity, and job satisfaction on these behaviors (MacKenzie et al., 1998), while neglecting the influence mechanisms of the new work context created by advancements in information technology, specifically, WCBA, on these behaviors. Based on the perspective of job resources and job demands, this research further examines psychological job control and ICT anxiety as a significant mechanism influencing both in-role and extra-role behaviors through work connectivity behavior. It enriches scholarly understanding of the links between WCBA and in-role and extra-role behaviors.

The second contribution is the exploration of the consequences of WCBA from the perspective of job resources and demands. Previous research mainly focuses on the single-factor impacts of WCBA on work–family conflicts (Yang et al., 2022), work and leisure (F. Wang et al., 2023), job satisfaction (Cheng et al., 2022), and psychological disturbances (Dong et al., 2022). This study utilized the JD-R model in the WCBA context and enriched the dual effect on employee behavior from positive and negative perspectives.

Third, the findings advance understanding of the association between WCBA and in-role and extra-role behaviors by evaluating the mediating effects of psychological job control and ICT anxiety using the JD-R model. From the perspective of the positive pathway, WCBA can be viewed as a job resource that enhances psychological job control. As a result, individual psychological resources are increased, significantly improving in-role and extra-role behaviors. From the negative pathway perspective, WCBA can be seen as a type of typical job demand outside regular work, triggering employees’ ICT anxiety. It hinders effective recovery, leading to further depletion of individual resources, and negatively impacting in-role and extra-role behaviors. This research explores the positive and negative effects of WCBA on these behaviors from an integrative perspective.

### 5.2. Practical Contributions

The findings have important practical implications for employees, organizations, and government. First, employees should fully leverage the positive effects of WCBA by enhancing their perceived control over work during non-working hours, thereby enhancing in-role and extra-role behaviors. For example, employees may benefit from developing effective time management strategies to enhance their sense of psychological job control while maintaining space for adequate rest and recovery. Tools such as task lists and calendar reminders may assist in organizing tasks more efficiently. Additionally, organizations should encourage employees to share experiences and updates on organizational dynamics and policies with colleagues through emails and phone calls. When employees maintain control over work outside of working hours, they can receive greater feedback, thus better fulfilling their job responsibilities and engaging in extra-role behaviors such as knowledge sharing and innovative practices. Considering that WCBA induces ICT anxiety among employees, leading to a decrease in in-role and extra-role behaviors, we recommend that employees who continue to focus on work-related information and tasks during non-working hours be cognizant of the negative effects of WCBA. Adopting reasonable relaxation methods, such as physical exercise and spending time with family and friends, can help individuals better regulate their emotions.

Second, organizations should pay attention to both sides of WCBA. Although our results demonstrate that WCBA has both positive and negative effects on employee behaviors, a comparative inspection of the path coefficients and indirect effects indicates that the positive mechanism through psychological job control exerts a greater impact than the negative mechanism through ICT anxiety. Thus, organizations may achieve net behavioral gains by promoting WCBA in a supportive and autonomy-enhancing context. On the one hand, organizations should fully recognize the positive effects of WCBA and adjust work mechanisms to meet employees’ psychological job control needs. This includes developing dedicated mobile applications or online platforms to facilitate employees in accessing work-related information, participating in discussions, and completing tasks during non-working hours. To avoid ambiguity, organizations should ensure that the use of WCBA-enabling platforms is voluntary and clearly regulated in order to prevent the risks of hyperconnectivity while maintaining the autonomy-enhancing benefits. Additionally, organizations can provide training and guidance to help employees acquire effective time management skills and work methods, thereby enhancing their work efficiency and quality during non-working hours. On the other hand, organizations should address the dark side of WCBA by providing psychological counseling and mind–body healing courses for employees to enhance their self-regulation abilities and alleviate ICT anxiety. Moreover, organizations should establish feedback mechanisms to promptly gauge employees’ perceptions of WCBA. Regular surveys and interviews can collect employees’ opinions and suggestions, enabling organizations to better understand the difficulties and challenges they face regarding WCBA, and ensuring effective control of the negative effects of WCBA.

Finally, the government should take the negative impact of WCBA on employees seriously and implement proactive measures to address it. This can be achieved by enacting relevant laws and regulations to standardize the management of non-working hours in organizations. By enacting legislation that defines employees’ rights during non-working hours and prohibits organizations from requiring work-related tasks during these times, the government can protect employees’ rights to rest and privacy. Additionally, the government should establish a supervisory mechanism to enhance oversight of organizational compliance with regulations on non-working hour management. By establishing specialized regulatory agencies or outsourcing to third-party organizations for regular inspections and assessments, the government can ensure that organizations comply with relevant laws and regulations and effectively safeguard the rights of employees. Furthermore, an employee assistance mechanism should be established to provide necessary support and assistance to employees affected by the negative impacts of WCBA. For example, setting up psychological counseling hotlines or providing mental health services contributes to helping employees alleviate work-related stress and anxiety.

### 5.3. Limitations and Future Research

Despite the potential usefulness of the study findings, several shortcomings remain, requiring further investigation. First, the study is cross-sectional. Although common method bias was not found to influence the findings, further longitudinal and field studies should be undertaken. Second, the sample exclusively comprised employees working in Chinese state-owned enterprises. Although this allows for greater control of organizational variability, it may limit the generalizability of the findings to other sectors or cultural contexts. In addition, the relatively high educational attainment of the sample may not fully represent the broader Chinese workforce. Future research could extend this work by comparing the effects of WCBA across organizational types (e.g., private firms, multinational corporations) and national cultures (e.g., countries differing on dimensions such as collectivism and power distance) to better understand the contextual boundaries of the dual-pathway model. Finally, this research did not differentiate between specific types of ICTs used (e.g., personal vs. professional devices), nor did it examine ICT factors such as system control, device ownership, or mandatory versus voluntary use. These contextual factors may shape user experiences, influencing the extent to which WCBA is perceived as a resource or a demand. For example, being connected after work via a personal device may increase perceived intrusion and ICT anxiety more than through the use of company-provided equipment. Future research should investigate how work versus non-work ICT use, as well as specific ICT features and affordances, may differentially influence psychological job control and ICT anxiety. Such investigations could provide a more granular understanding of how WCBA affects various work behaviors and further refine the theoretical boundaries of the dual-pathway model.

## Figures and Tables

**Table 1:** Sociodemographic characteristics of participants.

	%		%
Participant sex		Master’s degree and above	24.1
Male	38.8	Position	
Female	61.2	Ordinary employees	42.8
Age (years)		Front-line managers	30.3
18–25	18.2	Middle and senior managers	26.9
26–35	62.2	Tenure (years)	
36 and above	19.6	Less than 1	5.0
Education		1–3	22.6
Secondary school and below	2.2	4–6	28.9
College	6.7	7–10	20.9
Undergraduate degree	66.9	11 and above	22.6

Note(s): N = 402. Source(s): Table constructed by the authors from the primary data utilized in the analysis.

**Table 2:** Confirmatory factor analysis results.

Model	Factor	χ^2^	df	IFI	CFI	RMSEA	Model Comparison Test		
							Model Comparison	Δχ^2^	Δdf
1. Five-factor model	F1, F2, F3, F4, F5	921.739	530	0.947	0.946	0.043			
2. Four-factor model	F1, F2, F3, F4 + F5	2764.152	589	0.701	0.699	0.096	2 vs. 1	1842.413 ***	59
3. Three-factor model	F1, F2, F3 + F4 + F5	3455.647	593	0.606	0.604	0.110	3 vs. 1	2533.908 ***	63
4. Two-factor model	F1, F2 + F3 + F4 + F5	3860.81	596	0.551	0.549	0.117	4 vs. 1	2939.071 ***	66
5. One-factor model	F1 + F2 + F3 + F4 + F5	4222.994	598	0.501	0.499	0.123	5 vs. 1	3301.255 ***	68

Note(s): *N* = 402; F1 = work connectivity behavior after-hours; F2 = psychological job control; F3 = information and communication technology anxiety; F4 = in-role behaviors; F5 = extra-role behaviors; RMSEA = root mean square error of approximation; IFI = incremental fit index; CFI = comparative fit index. Source(s): Authors’ creation/work. *** *p* < 0.01.

**Table 3:** Means, standard deviations, correlations, and reliabilities of study variables.

Variable	Mean	Standard Deviation	1	2	3	4	5	6	7	8	9	10
1. Gender	1.610	0.488	1									
2. Age	2.070	0.729	−0.102 *	1								
3. Education	3.130	0.618	0.076	−0.191 **	1							
4. Position	1.840	0.820	−0.092	0.276 **	0.139 **	1						
5. Tenure	3.340	1.196	−0.161 **	0.746 **	−0.177 **	0.388 **	1					
6. WCBA	2.846	0.788	0.072	0.022	0.056	0.150 **	0.026	(0.782)				
7. Psychological job control	3.452	0.834	0.052	0.075	0.088	0.163 **	0.078	0.670 **	(0.813)			
8. ICT anxiety	1.998	0.782	0.110 *	−0.214 **	−0.048	−0.296 **	−0.321 **	0.113 *	−0.01	(0.832)		
9. In-role behaviors	4.071	0.661	0.017	0.003	0.065	0.096	0.034	0.609 **	0.596 **	−0.065	(0.844)	
10. Extra-role behaviors	4.08	0.648	0.013	0.053	0.049	0.101 *	0.116 *	0.449 **	0.443 **	−0.275 **	0.454 **	(0.911)

Note(s): *N* = 402; * *p* < 0.1, ** *p* < 0.05. Alpha reliabilities are presented in parentheses. Source(s): Authors’ creation/work.

**Table 4:** Path coefficient estimation results.

Path	Coefficient	S.E.	t	*p*
WCBA → Psychological job control	0.6975 ***	0.0398	17.5113	0.000
WCBA → ICT anxiety	0.1506 ***	0.0463	3.255	0.0012
Psychological job control → In-role behaviors	0.2735 ***	0.0407	6.7262	0.000
Psychological job control → Extra-role behaviors	0.1944 ***	0.046	4.2257	0.000
ICT anxiety → In-role behaviors	−0.1264 ***	0.0364	−3.4723	0.0006
ICT anxiety → Extra-role behaviors	−0.2882 ***	0.0378	−7.6236	0.000
WCBA → Extra-role behaviors	0.3669 ***	0.0372	9.8674	0.000
WCBA → In-role behaviors	0.5118 ***	0.0339	15.0775	0.000

Note(s): *** *p* < 0.01. Source(s): Authors’ creation/work.

**Table 5:** R^2^ values of models after controlling for sociodemographic variables.

Outcome Variable	Predictive Variable	R^2^	F	*p*
In-role behaviors	Psychological job control	0.3737	39.2875	0.0000
	WCBA			
In-role behaviors	ICT anxiety	0.3923	36.3400	0.0000
	WCBA			
Extra-role behaviors	Psychological job control	0.2165	18.1893	0.0000
	WCBA			
Extra-role behaviors	ICT anxiety	0.3172	26.1480	0.0000
	WCBA			

Source(s): Authors’ creation/work.

**Table 6:** Bootstrap analysis results.

Path	Effect	S.E.	Boot LLCI	Boot ULCI
WCBA–Psychological job control–In-role behaviors	0.1908	0.0365	0.1274	0.2724
WCBA–Psychological job control–Extra-role behaviors	0.1356	0.0445	0.591	0.2329
WCBA–ICT anxiety–In-role behaviors	−0.019	0.0093	−0.0407	−0.0045
WCBA–ICT anxiety–Extra-role behaviors	−0.0434	0.0142	−0.0731	−0.0174

Source(s): Authors’ creation/work.

## Data Availability

The raw data supporting the conclusions of this article will be made available by the authors upon request.
